# Inhibition of HDAC6 activity through interaction with RanBPM and its associated CTLH complex

**DOI:** 10.1186/s12885-017-3430-2

**Published:** 2017-07-01

**Authors:** Louisa M. Salemi, Matthew E. R. Maitland, Eyal R. Yefet, Caroline Schild-Poulter

**Affiliations:** Robarts Research Institute and Department of Biochemistry, Schulich School of Medicine & Dentistry, The University of Western Ontario, 1151 Richmond Street North, London, ON N6A 5B7 Canada

**Keywords:** RanBPM, HDAC6, α-tubulin, CTLH complex, Cell migration

## Abstract

**Background:**

Histone deacetylase 6 (HDAC6) is a microtubule-associated deacetylase that promotes many cellular processes that lead to cell transformation and tumour development. We previously documented an interaction between Ran-Binding Protein M (RanBPM) and HDAC6 and found that RanBPM expression inhibits HDAC6 activity. RanBPM is part of a putative E3 ubiquitin ligase complex, termed the C-terminal to LisH (CTLH) complex. Here, we investigated the involvement of the CTLH complex on HDAC6 inhibition and assessed the outcome of this regulation on the cellular motility induced by HDAC6.

**Methods:**

Cell lines (Hela, HEK293 and immortalized mouse embryonic fibroblasts) stably or transiently downregulated for several components of the CTLH complex were employed for the assays used in this study. Interactions of HDAC6, RanBPM and muskelin were assessed by co-immunoprecipitations. Quantifications of western blot analyses were employed to evaluate acetylated α-tubulin levels. Confocal microscopy analyses were used to determine microtubule association of HDAC6 and CTLH complex members. Cell migration was evaluated using wound healing assays.

**Results:**

We demonstrate that RanBPM-mediated inhibition of HDAC6 is dependent on its association with HDAC6. We show that, while HDAC6 does not require RanBPM to associate with microtubules, RanBPM association with microtubules requires HDAC6. Additionally, we show that Twa1 (Two-hybrid-associated protein 1 with RanBPM) and MAEA (Macrophage Erythroblast Attacher), two CTLH complex members, also associate with α-tubulin and that muskelin, another component of the CTLH complex, is able to associate with HDAC6. Downregulation of CTLH complex members muskelin and Rmnd5A (Required for meiotic nuclear division homolog A) resulted in decreased acetylation of HDAC6 substrate α-tubulin. Finally, we demonstrate that the increased cell migration resulting from downregulation of RanBPM is due to the relief in inhibition of HDAC6 α-tubulin deacetylase activity.

**Conclusions:**

Our work shows that RanBPM, together with the CTLH complex, associates with HDAC6 and restricts cell migration through inhibition of HDAC6 activity. This study uncovers a novel function for the CTLH complex and suggests that it could have a tumour suppressive role in restricting HDAC6 oncogenic properties.

**Electronic supplementary material:**

The online version of this article (doi:10.1186/s12885-017-3430-2) contains supplementary material, which is available to authorized users.

## Background

Ran binding protein M (RanBPM), also referred to as RanBP9, is a 90 kDa ubiquitous protein localized both in the nucleus and the cytoplasm which has been implicated in various cellular functions but has no intrinsic enzymatic activity. RanBPM contains a Spla kinase and ryanodine receptor (SPRY) domain, known to mediate protein-protein interactions. It also contains a lissencephaly type-1 like homology (LisH) domain, known to mediate protein dimerization and tetramer formation and a C-terminal to LisH (CTLH) domain, whose function remains unknown [1–3]. The C terminal CT-11-RanBPM (CRA) domain is made up of six helices that resemble the death domain superfamily [4]. RanBPM has been shown to interact with numerous proteins, implicating RanBPM to function in a variety of cellular processes including cell adhesion, migration, microtubule dynamics, and gene transcription [5–7]. It has been hypothesized that RanBPM functions as a scaffolding protein that is part of a large complex [5, 6, 8, 9]. RanBPM is well conserved in eukaryotes and a RanBPM counterpart, glucose-induced degradation-deficient 1 (Gid1), also called vacuolar import and degradation 30 (Vid30), has been identified in yeast [10, 11]. Gid1 also contains SPRY, LisH, CTLH and CRA domains and is a component of a large protein complex made up of several other Gid proteins [5, 10–12]. The mammalian homologs of almost all of these proteins have also been found in a large complex, which has been called the CTLH complex [13]. The CTLH complex also includes the protein muskelin, which is not encoded in the yeast genome [13]. The yeast complex functions as an E3 ubiquitin ligase in the degradation of fructose-1,6-bisphosphatase (FBPase), a gluconeogenic enzyme required when yeast cells are grown in a carbon-poor medium [5, 10, 11]. The Really Interesting New Gene (RING) domains of Gid2 and Gid9 which confer the E3 ubiquitin ligase activity in the Gid complex are conserved in the human homologs required for meiotic nuclear division homolog A (Rmnd5A) and macrophage erythroblast attacher (MAEA), respectively [11, 13, 14]. This suggests that the human CTLH complex may also have E3 ubiquitin ligase activity, however this remains to be demonstrated.

Our previous studies have suggested that RanBPM has tumour-suppressive activities. Downregulation of RanBPM resulted in decreased apoptotic activation in response to ionizing radiation (IR) and caused increased expression of Bcl-2, an anti-apoptotic Bcl-2 family member [2]. Downregulation of RanBPM also resulted in loss of growth factor dependence and in increased cell motility, suggesting that RanBPM expression confers activities that restrict cell growth and cell migration [15].

Histone deacetylase 6 (HDAC6) is a class IIb HDAC and, unlike other HDAC enzymes, HDAC6 shows cytoplasmic localization. HDAC6 uniquely has duplicate deacetylase domains as well as a C-terminal binder of ubiquitin zinc finger (BUZ) domain, which is able to bind ubiquitin [16]. Although named HDAC6, it does not have detectable deacetylase activity toward histones in vivo [17, 18]. Its most characterized substrates include α-tubulin, heat shock protein 90 (Hsp90) and cortactin [17]. HDAC6 deacetylation activity is both negatively and positively regulated by post-translational modifications. Several kinases, such as protein kinase C (PKC) ζ, PKCα, G protein-coupled receptor kinase 2 (GRK2), glycogen synthase kinase 3 β (GSKβ), casein kinase 2 (CK2), ERK and Aurora A phosphorylate HDAC6 and promote HDAC6 α-tubulin deacetylase activity [19–25]. Conversely, epidermal growth factor receptor (EGFR) phosphorylation of HDAC6 decreases tubulin deacetylase activity [26]. Acetylation of HDAC6 by p300 also inhibits HDAC6 α-tubulin deacetylase activity [27]. In addition to being regulated by post-translational modification, HDAC6 is also regulated by protein-protein interactions. HDAC6 association with dysferlin, p62, paxillin, tau and tubulin polymerization-promoting protein/p25 (TPPP/p25) result in decreased α-tubulin deacetylase activity [28–32].

HDAC6 associates with microtubules and has been shown to promote cell motility through deacetylation of α-tubulin and/or cortactin [18, 33, 34]. Overexpression of HDAC6 results in increased cell motility and knockout, downregulation or inhibition of HDAC6 by trichostatin A (TSA) or tubacin results in severely reduced cell migration [18, 33–37]. This clearly indicates a role for HDAC6 catalytic activity in promoting cell motility.

HDAC6 has been implicated in cancer development and HDAC6-specific inhibitors have emerged as a chemotherapeutic agent to combat cancer. HDAC6 is required for in vitro oncogene-induced cell transformation and transforming growth factor (TGF) β1 induced epithelial-mesenchymal transition (EMT) [38, 39]. HDAC6 expression is also required to maintain anchorage-independent growth of established cancer cell lines [38]. HDAC6 has been shown to promote tumour formation in mouse models [38]. Upregulated HDAC6 levels have been observed in many cancer cell lines and in cohorts of oral squamous cell carcinoma (OSCC) and hepatocellular carcinoma (HCC) patients [40, 41]. HDAC6 has also been demonstrated to play a role in promoting angiogenesis [42, 43]. Several clinical trials are in progress using ACY-1215, an HDAC6 specific inhibitor alone or in combination with other agents. Preclinical studies for ACY-1215 have shown very promising results for the treatment of multiple myeloma, non-Hodgkin lymphoma and inflammatory breast cancer and the treatment was well tolerated in animals [44–46].

We have previously demonstrated that RanBPM is able to form a complex with HDAC6 [47]. The LisH/CTLH domains of RanBPM were found necessary for association with HDAC6, as deletion of these domains resulted in loss of interaction [47]. We also reported that RanBPM is able to inhibit HDAC6 activity using an in vitro HDAC6 activity assay. Consistent with this, levels of acetylated α-tubulin, a specific HDAC6 substrate were found significantly reduced in cells stably expressing RanBPM shRNA compared to those expressing a control shRNA [47]. Interestingly, we also reported that RanBPM partially co-localizes with microtubules in both Hela and 3T3 mouse embryonic fibroblasts (MEFs) and that RanBPM associates with α-tubulin using co-immunoprecipitation [48].

In this study, we show that RanBPM-mediated inhibition of HDAC6 α-tubulin deacetylase activity is dependent on its association with HDAC6. The RanBPM-HDAC6 interaction requires the second catalytic domain of HDAC6 and the LisH domain of RanBPM. We show that HDAC6 does not require RanBPM to associate with microtubules, but that RanBPM colocalization to microtubules requires HDAC6. Furthermore, we demonstrate that components of the CTLH complex associate with both microtubules and HDAC6 and that muskelin, another component of the CTLH complex, associates with HDAC6 and mediates HDAC6 inhibition. Lastly, RanBPM was found to inhibit HDAC6 mediated cell migration. Our work suggests that RanBPM, together with the CTLH complex, associates with HDAC6 and inhibits HDAC6 activities.

## Methods

### Plasmid expression constructs

pCMV-HA RanBPM shRNA mutant construct (WT RanBPM) and pCMV-HA-RanBPM-Δ360 (Δ360), pCMV-HA-RanBPM-ΔLisH (ΔLisH) and pCMV-HA-RanBPM-ΔCTLH (ΔCTLH) were previously described [2, 47, 48]. pcDNA-HDAC6-FL-FLAG (FL) (Addgene Plasmid #30482), pcDNA-HDAC6-DC-FLAG (DC) (Addgene Plasmid #30483) were obtained from Addgene and pcDNA-HDAC6–1-840-FLAG (1–840), pcDNA-HDAC6–1-503-FLAG (1–503) and pcDNA-HDAC6-ΔN-439-1215-FLAG (ΔN-439) were a gift from Tso-Pang Yao [49]. pcDNA-HA-HDAC6–1-840-FLAG (HA-1-840) and pcDNA-HA-HDAC6–1-503-FLAG (HA-1-503) constructs were produced using annealed HA-tag oligonucleotides that generated overhangs that could be ligated with digested pcDNA-HDAC6–1-840-FLAG (1–840) and pcDNA-HDAC6–1-503-FLAG (1–503), respectively. pGEX4T1-GST-WT-RanBPM was generated by PCR amplification of full length RanBPM from pCMV-HA-RanBPM and cloned into digested pGEX4T1. pET28a-HDAC6-catalytic domain 2 (CAT2) was generated by PCR amplification of the second catalytic domain of HDAC6 from pcDNA-HDAC6-FL-FLAG and cloned into digested pET28a. All PCR reactions were done using KOD polymerase (Novagen, Germany) and primers from Integrated DNA Technologies (Coralville, Iowa, USA).

### Cell culture, transfections and treatments

Hela, Hela control and RanBPM shRNA cells, HEK293, HEK293 control and RanBPM shRNA cells were previously described [2, 15]. Wildtype (WT) and HDAC6 knockout (KO) MEFs were a gift from Tso-Pang Yao [36]. All cells were cultured in high glucose Dulbecco’s modified Eagle’s medium (DMEM) supplemented with 10% fetal bovine serum (10%) at 37 °C in 5% CO_2_. Control and RanBPM shRNA stable Hela and HEK cells were maintained in media supplemented with 0.35 mg/ml and 0.45 mg/ml G418, respectively (Geneticin, Bioshop Canada, Burlington, ON Canada). Tubacin (Cayman Chemical) was added to the cell media at the concentrations and durations indicated in the figure legends.

### Transfection assays

Plasmid transfections were carried out with jetPRIME (Polypus Transfection) according to the manufacturer’s protocol or by calcium phosphate transfection according to standard protocols. siRNAs directed against Muskelin (Silencer Select, s8799, Ambion), Rmnd5A (Silencer, 125,392, Ambion), and a negative control siRNA (Silencer Select, cat# 4390843, Ambion) were purchased from ThermoFisher Scientific. siRNAs were transfected into HeLa cells seeded on a 60 mm dish using JetPRIME (Polyplus-transfection) at a final concentration of 60 nM. After 72 h, extracts were prepared and remaining cells were passaged onto a 100 mm plate and cultured for an additional 72 h.

### Extract preparation, western blot and immunoprecipitations

Whole cell extracts were prepared as described [2] and resolved by SDS-PAGE (between 8% and 12%) and transferred to polyvinylidene difluoride (PVDF) membranes. Samples were analyzed with the following antibodies: HDAC6 (H-300, Santa Cruz, Santa Cruz, CA, USA, and PA5–11240, ThermoFischer Scientific), HA (HA-7, Sigma–Aldrich), Acetylated α-tubulin (6–11B-1, Santa Cruz, Santa Cruz, CA, USA), α-tubulin (T5168, Sigma–Aldrich), β-Actin (I-19, Santa Cruz, Santa Cruz, CA, USA), RanBPM (5 M, Bioacademia, Japan), Rmnd5A (NBP1–92337, Novus Biologicals) and muskelin (C-12, Santa Cruz, Santa Cruz, CA, USA). The blots were developed using Clarity ECL Western Blotting Substrate (BioRad, Hercules, CA). Quantifications were done using Image Lab (BioRad, Hercules, CA) and ImageJ software. Co-immunoprecipitation experiments were performed in 0.25% NP-40 and 100 mM KCl lysis buffer and were carried out overnight at 4 °C with antibodies to HA (HA-7, Sigma–Aldrich), OctA-Probe (D-8 Santa Cruz, Santa Cruz, CA, USA), HDAC6 (D-11 Santa Cruz, Santa Cruz, CA, USA) and RanBPM (F1 Santa Cruz, Santa Cruz, CA, USA). Immunoprecipitates were isolated with PureProteome Protein G Magnetic Beads (EMD Millipore, Billerica, Massachusetts) or Dynabeads Protein G (Invitrogen, Life Technologies, Burlington ON, Canada).

### Immunofluorescence and confocal microscopy

Cells were plated on coverslips and following overnight incubation were either fixed or transfected and incubated for 24 h. Cells were fixed with 3% paraformaldehyde, permeabilized in 0.5% Triton-X100 for 10 min and pre-blocked in 5% FBS diluted in PBS. Coverslips were incubated overnight with primary antibodies (see below), washed in PBS and incubated with secondary antibodies: anti-rabbit Alexa Fluor 488, anti-goat Alexa Fluor 488, anti-mouse Alexa Fluor 488, anti-mouse Alexa Fluor 647 and anti-rabbit Alexa Fluor 647. Cells were mounted with ProLong Gold antifade with DAPI (Invitrogen). Primary antibodies used in immunofluorescence: HDAC6 (H-300, Santa Cruz, Santa Cruz, CA, USA), α-tubulin (T5168, Sigma–Aldrich), RanBPM (K12, Santa Cruz, Santa Cruz, CA, USA), HA (HA-7, Sigma–Aldrich), α-tubulin (ab15246, Abcam), MAEA (ab151304, Abcam) and Twa (ab97653, Abcam). Confocal images were acquired using an inverted IX51 Olympus microscope equipped with a Perkin Elmer Spinning Disk confocal attachment with a 60× objective using Velocity software (Improvision). Z-stack image deconvolution was done using AutoQuant (Media Cybernetics, Rockville, MD, USA) software and image analyses for both plane and Z-stacks images were done using Imaris software (Bitplane, Zurich, Switzerland). Colocalization analyses were done using Imaris software using the top 2% of colocalized voxels.

### Scratch assay

Scratch assay experiments were performed as described [15]. Briefly, control and RanBPM shRNA HEK cells were plated and following overnight incubation were transfected with pCMV empty vector (EV), pCMV-HA-RanBPM-WT (WT) or pCMV-HA-RanBPM-Δ360 (Δ360). Cell monolayers were incubated in the presence of 2 mM hydroxyurea (Sigma-Aldrich) for 24 h to prevent cell proliferation. Cells were scratched using a sterile 200 μL pipette tip following a 4 h treatment with either DMSO or tubacin. Wound closure was assessed at 0 h and 24 h using a fluorescent microscope (IX70, Olympus) and images were captured using a charge-coupled device camera (Q-imaging). Samples were performed in triplicate, three pictures were taken per sample, and the wound width was measured using ImageJ software using an average of three width measurements per picture. Fold migration was calculated by normalizing the average wound width at 24 h to the average wound width at 0 h.

### Statistical analysis

Differences between multiple groups were compared using analysis of variance (ANOVA) and differences between two groups were compared using unpaired two-tailed t test. Results were considered significant when *P* < 0.05.

## Results

### HDAC6 and RanBPM interaction

**Fig. 1:**
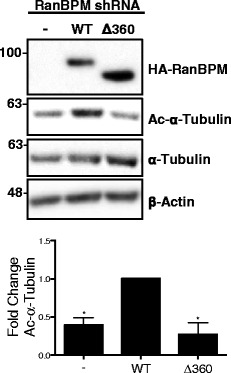
RanBPM association with HDAC6 is required for inhibition of HDAC6 deacetylase activity. *Top*, whole cell extracts from RanBPM shRNA Hela cells either left untransfected or transfected with HA-RanBPM-WT or HA-RanBPM-Δ360 were analyzed by western blot with the antibodies indicated. *Bottom*, quantification of relative amounts of acetylated α-tubulin was normalized to total α-tubulin levels. Relative amounts of acetylated α-tubulin in untransfected and HA-RanBPM-Δ360-transfected cells were normalized to WT. Results are averaged from three different experiments, with error bars indicating SEM. *P* < 0.05 (*)

We previously documented that RanBPM shRNA cells display decreased levels of acetylated α-tubulin compared to control shRNA cells, and that these levels could be restored to that of control cells upon re-expression of RanBPM, demonstrating that RanBPM expression inhibits HDAC6 activity [47]. To determine if RanBPM association with HDAC6 is necessary for its inhibition, we assayed levels of acetylated α-tubulin in RanBPM shRNA cells re-expressing either wildtype RanBPM or a RanBPM mutant bearing a LisH/CTLH domain deletion which we previously demonstrated impairs RanBPM’s association with HDAC6 [47]. We found that cells transfected with the LisH/CTLH (Δ360) RanBPM mutant had levels of acetylated α-tubulin similar to that of RanBPM shRNA cells and therefore was not able to restore acetylated α-tubulin to the same level as wildtype RanBPM (Fig. 1). This indicates that association of RanBPM through the LisH and/or CTLH domains is required for inhibition of HDAC6.

**Fig. 2:**
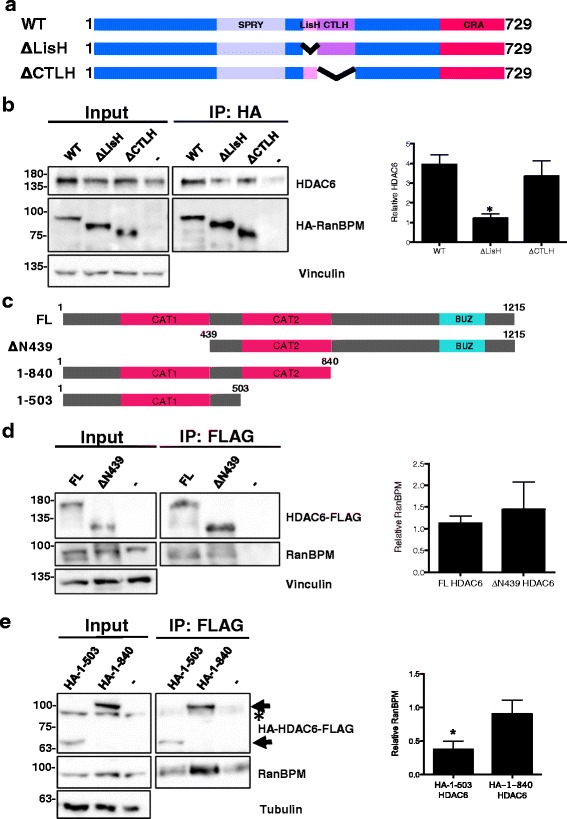
Identification of the RanBPM and HDAC6 domains that mediate their association. **a** Schematic representation of HA-tagged RanBPM wildtype (WT) and deletion mutant constructs ΔLisH and ΔCTLH. **b** *Left,* whole cell extracts were prepared from RanBPM shRNA Hela cells untransfected (−) or transfected with HA-RanBPM-WT, HA-RanBPM-ΔLisH, or HA-RanBPM-ΔCTLH constructs. RanBPM was immunoprecipitated with an HA antibody and immunoprecipitates were analyzed by western blot with HDAC6 and RanBPM antibodies. Input, 5% input extract. *Right,* quantification of relative amounts of co-immunoprecipitated HDAC6 normalized to immunoprecipitated RanBPM. Results are averaged from three different experiments with error bars indicating SEM. *P* < 0.05 (*). **c** Schematic representation of HDAC6 full length (FL) and deletion mutant constructs*.* **d** *Left,* whole cell extracts were prepared from HDAC6 knockout MEFs untransfected (−) or transfected with full length (FL) or ΔN439 HDAC6 constructs. HDAC6 was immunoprecipitated with a FLAG antibody and immunoprecipitates were analyzed by western blot with RanBPM and HDAC6 antibodies. Input, 5% input extract. *Right,* quantification of relative amounts of co-immunoprecipitated RanBPM normalized to immunoprecipitated HDAC6. Results are averaged from three different experiments with error bars indicating SEM. *P* < 0.05 (*). **e** *Left,* whole cell extracts were prepared from HDAC6 knockout MEFs untransfected (−) or transfected with HA-1-503 or HA-1-840 HDAC6 constructs and immunoprecipitation and analysis was performed as described above except that HDAC6 mutant immunoprecipitation was verified using an HA antibody. Arrows indicate the position of the HA-HDAC6 mutants. The asterisk indicates residual RanBPM signal from previous hybridization. *Right,* quantification of relative amounts of co-immunoprecipitated RanBPM normalized to immunoprecipitated HDAC6. Results are averaged from three different experiments with error bars indicating SEM. *P* < 0.05 (*)

Since complex formation between RanBPM and HDAC6 had an important effect on HDAC6 activity, we investigated in more detail the specifics of this interaction. To determine more precisely the RanBPM domain responsible for association with HDAC6, we generated individual deletions of the LisH and CTLH domains and by co-immunoprecipitation analysis we assayed their ability to associate with HDAC6. Deletion of the LisH domain resulted in significantly reduced co-immunoprecipitation of HDAC6, whereas deletion of the CTLH did not affect HDAC6 association (Fig. 2a and b). These results indicate that the LisH domain is necessary for the interaction of RanBPM with HDAC6. To confirm the importance of the RanBPM LisH domain in modulating HDAC6 activity, we assessed the effect of the LisH domain deletion on RanBPM’s ability to restore acetylated α-tubulin levels upon transfection in RanBPM shRNA cells (Additional file 1: Fig. S1). As expected, RanBPM LisH deletion mutant had levels of acetylated α-tubulin similar to that of RanBPM shRNA cells, confirming that the LisH domain is necessary for the inhibitory effect of RanBPM on HDAC6 activity.

To evaluate which region of HDAC6 is required for association with RanBPM, we transfected HA and/or FLAG tagged HDAC6 deletion constructs (Fig. 2c) [49] into HDAC6 knockout (KO) mouse embryonic fibroblasts (MEFs) and performed co-immunoprecipitation analyses. Deletion of the N-terminal region of HDAC6 (ΔN439) did not affect HDAC6 association with RanBPM (Fig. 2d), indicating that the N-terminal region of HDAC6 is not required for association with RanBPM. HDAC6 deletion mutant 1–840, which lacks HDAC6 C-terminal domain but contains both catalytic domains, retained its ability to interact with RanBPM. However deletion mutant 1–503, which only contains the first catalytic domain, was no longer able to associate with RanBPM (Fig. 2e). This indicates that the second catalytic domain of HDAC6 is the region responsible for the interaction with RanBPM.

We then investigated if the association of RanBPM and HDAC6 was direct. We generated constructs to bacterially express full length RanBPM and an HDAC6 peptide corresponding to the second catalytic domain of HDAC6. We performed GST-pull-down assays where GST-tagged RanBPM was incubated with T7-tagged HDAC6 catalytic domain 2 (CAT2). However, we were unable to detect HDAC6 CAT2 in the GST-RanBPM pull-downs, suggesting that the interaction of HDAC6 and RanBPM is not direct (Additional file 2: Fig. S2).

### RanBPM association with α-tubulin

**Fig. 3:**
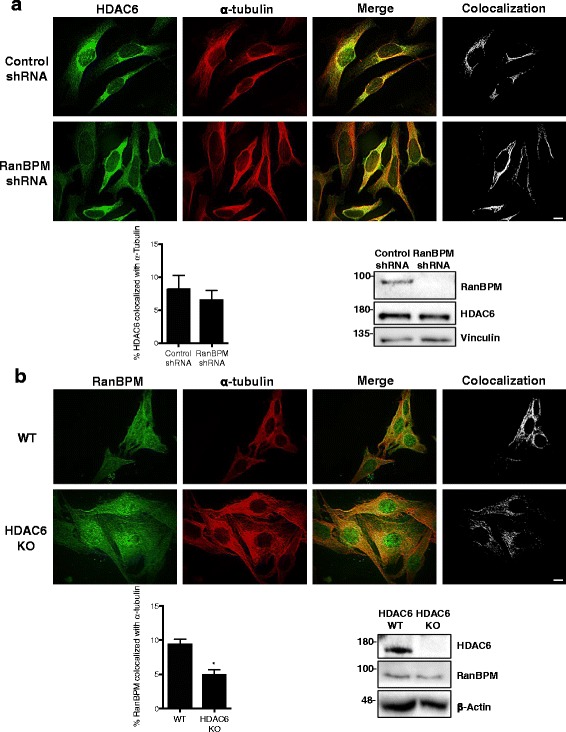
RanBPM requires HDAC6 for association with α-tubulin. **a** Control or RanBPM shRNA Hela cells were fixed and incubated with antibodies against HDAC6 (green) and α-tubulin (red). Colocalization of HDAC6 and α-tubulin was analyzed using Imaris software. *Top,* representative images where white signal represents the top 2% of HDAC6 and α-tubulin colocalization. *Bottom left,* quantification of the top 2% of pixels representing HDAC6 colocalized with α-tubulin. Data are representative of a minimum of 60 cells from three experiments. Error bars represent SEM. *P* < 0.05 (*). *Bottom right,* western blot analysis of extracts from Hela control and RanBPM shRNA cells, showing RanBPM and HDAC6 expression with respect to vinculin loading control. **b** Wildtype (WT) or HDAC6 knockout (KO) MEFs were fixed and incubated with antibodies against RanBPM (green) and α-tubulin (red). Colocalization of RanBPM and α-tubulin was analyzed using Imaris software. *Top,* representative images where white signal represents the top 2% of RanBPM and α-tubulin colocalization. *Bottom left,* quantification of the top 2% of pixels representing RanBPM colocalized with α-tubulin. Data are representative of a minimum of 60 cells from three experiments. Error bars represent SEM. *P* < 0.05 (*). *Bottom right,* western blot analysis of extracts from WT and HDAC6 KO MEFs, showing HDAC6 and RanBPM expression with respect to β-actin loading control

We have previously established that RanBPM colocalizes with microtubules and associates with α-tubulin [48]. Since HDAC6 associates with α-tubulin [17], we wanted to determine whether RanBPM was necessary for HDAC6 association with α-tubulin and vice versa. First, we assayed whether HDAC6 could associate with α-tubulin independently of RanBPM using quantitative measurements of confocal microscopy analysis of HDAC6 colocalization with α-tubulin in control or RanBPM shRNA cells (Fig. 3a). We found no significant difference in the amount of HDAC6 colocalized with α-tubulin in control and RanBPM shRNA cells, indicating that HDAC6 is able to associate with α-tubulin independent of RanBPM. Next, we performed the reciprocal experiment to determine if RanBPM colocalization with α-tubulin was dependent on HDAC6 using wildtype (WT) and HDAC6 knockout (KO) MEFs. For this experiment, colocalization analysis indicated a significant decrease in RanBPM colocalized with α-tubulin in HDAC6 KO cells (Fig. 3b), denoting that RanBPM requires HDAC6 for association with α-tubulin. Altogether, this suggests that RanBPM does not directly interact with microtubules and that HDAC6 is required to mediate RanBPM association with microtubules.

**Fig. 4:**
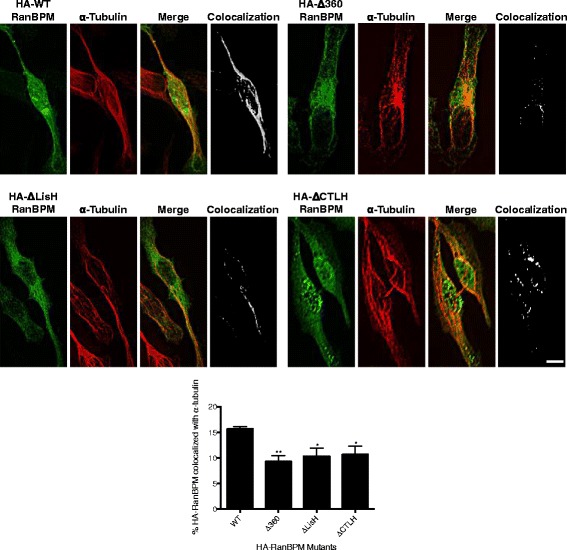
RanBPM requires the LisH and CTLH domains to associate with α-tubulin. Hela RanBPM shRNA cells were transfected with the indicated HA-RanBPM deletion constructs. Cells were fixed and incubated with antibodies against HA (green) and α-tubulin (red). Colocalization of HA-RanBPM and α-tubulin was analyzed using Imaris software. *Top,* representative images where white signal represents the top 2% of HA-RanBPM and α-tubulin colocalization. Scale bar: 10 μm. *Bottom,* Quantification of the top 2% of pixels representing HA-RanBPM colocalized with α-tubulin. Data are representative of a minimum of 10 transfected cells from three experiments. Error bars represent SEM. *P* < 0.05 (*)

The observation that RanBPM colocalization with microtubules was dependent on HDAC6 expression suggested that RanBPM association with HDAC6 was necessary for its colocalization with microtubules, specifically α-tubulin. To assess this, we transfected HA-tagged WT, Δ360, ΔLisH and ΔCTLH RanBPM constructs into RanBPM shRNA cells and assessed colocalization of the WT and RanBPM mutants and α-tubulin using confocal microscopy analysis. Intriguingly, we found significantly reduced association of all three RanBPM mutants with α-tubulin when compared to wildtype (Fig. 4). As we have previously shown that the RanBPM LisH domain is required for association with HDAC6 and that HDAC6 is necessary for RanBPM to associate with α-tubulin, it was expected that Δ360 and ΔLisH would have reduced association with α-tubulin. However, since the ΔCTLH HA-tagged RanBPM also demonstrated significantly reduced colocalization with α-tubulin, this suggests that the CTLH domain is also involved in RanBPM’s association with α-tubulin, independent of HDAC6 association.

### HDAC6 association with the CTLH complex

**Fig. 5:**
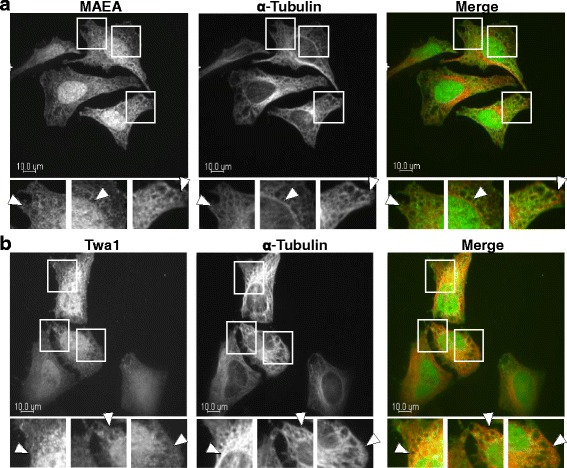
CTLH components associate with microtubules. **a** Hela cells were fixed and incubated with antibodies against MAEA and α-tubulin. Shown are single plane confocal images. Insets are enlarged images of the boxed regions from the above *panels* and *arrows* indicate areas of colocalization. The* right panels* show merged images (MAEA, *green*; α-tubulin, *red*) Scale bar: 10 μm. **b** Hela cells were fixed and incubated with antibodies against Twa1 and α-tubulin and analysis was performed as described above (Twa1, *green*; α-tubulin, *red*)

As RanBPM is known to be a component of the CTLH complex, our next objective was to determine whether other members of the CTLH complex could also associate with HDAC6. We first evaluated whether components of the CTLH complex were able to colocalize with α-tubulin as we have previously shown for RanBPM. In Hela cells, we established that both endogenous MAEA and Twa1, two members of the CTLH complex, colocalized with α-tubulin by confocal microscopy analysis (Fig. 5), indicating that the CTLH complex as a whole is present at microtubules.

**Fig. 6:**
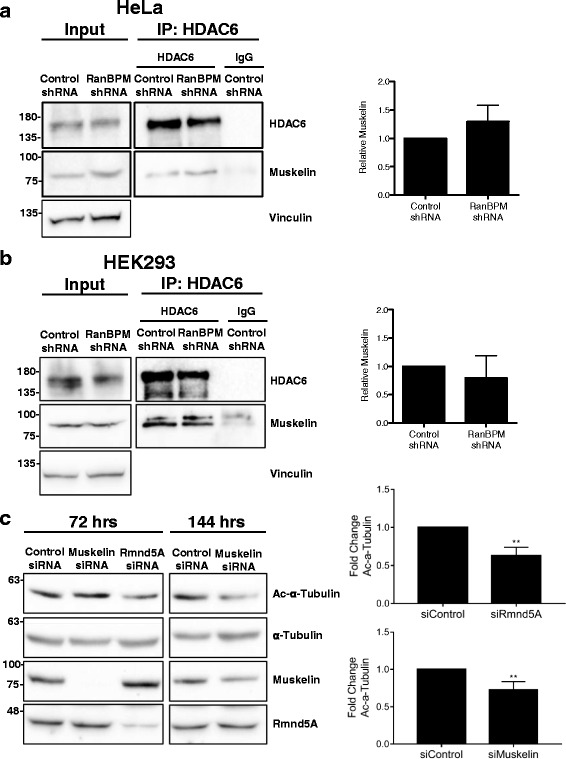
Muskelin associates with HDAC6 and downregulation of Rmnd5A and muskelin affects α-tubulin acetylation. **a** HDAC6 associates with Muskelin independently of RanBPM. *Left,* whole cell extracts were prepared from control or RanBPM shRNA Hela cells and immunoprecipitated with either HDAC6 or IgG. Immunoprecipitates were analyzed by western blot with a muskelin antibody. HDAC6 immunoprecipitation was verified using an HDAC6 antibody. Input, 5% input extract. *Right,* quantification of relative amounts of co-immunoprecipitated muskelin normalized to immunoprecipitated HDAC6. Results are averaged from three different experiments with error bars indicating SEM. *P* < 0.05 (*). **b** Whole cell extracts were prepared from Control or RanBPM shRNA HEK cells and analyzed as described above. **c** Whole cell extracts were prepared from HeLa cells transfected with control, Rmnd5A, or Muskelin siRNA. Extracts were prepared either 72 or 144 h after transfection and analyzed by western blot with the indicated antibodies. *Right*, quantification of relative amounts of acetylated α-tubulin normalized to total α-tubulin levels. Results are averaged from at least three different experiments, with *error bars* indicating SD. *P* < 0.005 (**)

As we could not identify a direct interaction between HDAC6 CAT2 and RanBPM in our in vitro binding assays (Additional file 2: Fig. S2), we speculated that another member of the CTLH complex could be directly associating with HDAC6 to mediate the interaction between HDAC6 and RanBPM. It came to our attention that muskelin, a component of the CTLH complex has been shown to interact with HDAC6 in a large proteomic screen [50]. Muskelin interacts with RanBPM [6, 51] and interestingly, muskelin, through its LisH and CTLH domains, has previously been shown to interact directly with the retrograde microtubule motor protein dynein [52]. Thus we employed a co-immunoprecipitation analysis to evaluate if muskelin could associate with HDAC6 and whether this association was dependent on RanBPM. HDAC6 was immunoprecipitated from both control and RanBPM shRNA Hela and HEK293 cells and the association of muskelin was evaluated by western blot. Muskelin was co-immunoprecipitated with HDAC6 to similar levels in both control and RanBPM shRNA cells (Fig. 6a, b), indicating that muskelin does not require RanBPM to associate with HDAC6. Thus, muskelin associates with HDAC6 independent of RanBPM, suggesting that it could bridge RanBPM to HDAC6. Finally, to assess the involvement of the CTLH complex in HDAC6 inhibition, we evaluated acetylated α-tubulin levels in cells transiently transfected with siRNAs against muskelin and Rmnd5A. Downregulation of both CTLH complex members resulted in significantly decreased acetylated α-tubulin levels indicative of increased HDAC6 activity (Fig. 6c). Interestingly, these effects occurred at different time points following siRNA transfection (muskelin, 144 h and Rmnd5A, 72 h), which could be explained by differences in the stability of these two proteins, and/or reflect a different effect of their downregulation on the CTLH complex. Overall, our results show that various components of the CTLH complex associate with HDAC6 and affect HDAC6 activity, suggesting that this reflects a regulation of HDAC6 by the entire CTLH complex.

### RanBPM inhibits HDAC6-mediated cell migration

**Fig. 7:**
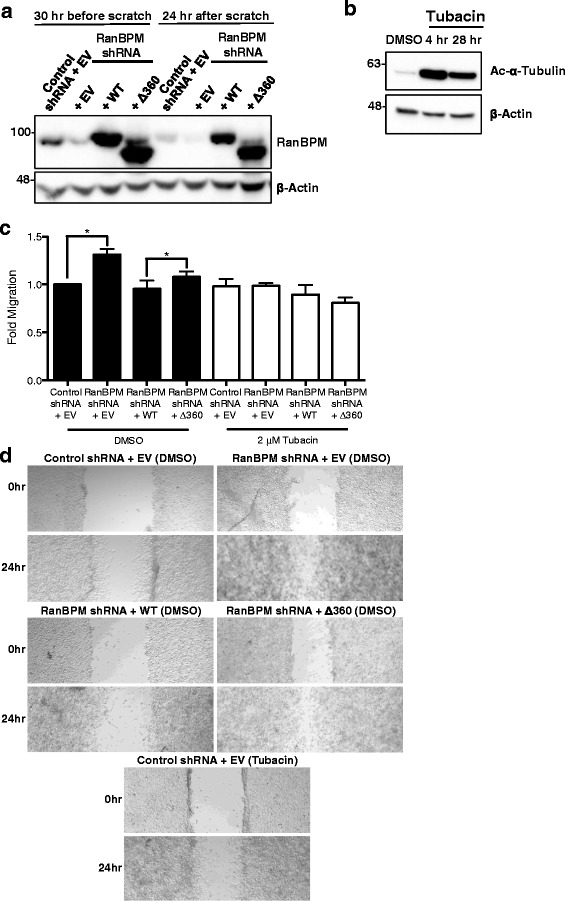
RanBPM inhibits HDAC6 mediated cell migration. **a** Control or RanBPM shRNA HEK293 cells transfected with either empty vector (EV), WT-RanBPM (WT) or Δ360 RanBPM (Δ360) were harvested 30 h before the scratch or 24 h after the scratch. Whole cell extracts were analyzed by western blot and hybridized with the antibodies indicated. **b** Control shRNA HEK293 cells were treated with either DMSO or 2 μM Tubacin for the indicated time points. Whole cell extracts were analyzed by western blot and hybridized with the antibodies indicated. **c** Confluent monolayers of control or RanBPM shRNA HEK293 cells were transfected with either EV, WT or Δ360 RanBPM and were cultured in the presence of 2 mM hydroxyurea for 24 h, and pretreated with either DMSO (black bars) or 2 μM Tubacin (*white bars*) for 4 h. Cells were then scratched and width of the wound was assessed at the time of the scratch and 24 h later. Fold migration was calculated by normalizing the average wound width at 24 h to the average wound width at 0 h and each sample was normalized to control shRNA EV transfected, DMSO treated samples. Results are averaged from three independent experiments, with *error bars* indicating SEM. *P* < 0.05 (*). **d** Representative images of Control or RanBPM shRNA HEK cells transfected and treated as above following the scratch (0 h) and 24 h later

To evaluate the functional effects of RanBPM inhibition of HDAC6 on cell migration, we performed a wound healing assay, also called scratch assay [53]. A confluent monolayer of control shRNA cells transfected with empty vector (EV) or RanBPM shRNA cells transfected with either EV, WT or Δ360 RanBPM were incubated for 24 h with hydroxyurea to prevent cell proliferation. To confirm constant expression of mutant RanBPM constructs throughout the assay, an extract was prepared 24 h after transfection and at the end of the assay (24 h after the scratch) (Fig. 7a). Prior to the scratch, cells were pretreated for 4 h with either DMSO or tubacin, a specific HDAC6 inhibitor [37]. Four-hour treatment with tubacin was shown to result in increased acetylated α-tubulin which persisted up to 28 h post treatment, indicating that HDAC6 α-tubulin deacetylase activity is inhibited throughout the duration of the assay (Fig. 7b) [35]. Following the scratch, cells were imaged and the width of the scratch was measured at time zero and again twenty-four hours later. As previously reported [15], RanBPM shRNA cells showed significantly increased cell migration compared to control shRNA (Fig. 7c). Reintroduction of WT RanBPM was able to decrease cell migration to that of control cells. However, the Δ360 RanBPM mutant, which does not associate with HDAC6 and is unable to inhibit HDAC6 α-tubulin deacetylase activity, still displayed significantly increased cell migration compared to WT RanBPM suggesting that this mutation impaired its ability to regulate cell motility (Fig. 7c and d). Tubacin treatment of all conditions resulted in cell migration comparable to that of control shRNA, indicating that increased cell migration in RanBPM shRNA cells is due to the relief of RanBPM-mediated inhibition of HDAC6 α-tubulin deacetylase activity.

## Discussion

Expanding on our previous findings that RanBPM is able to inhibit HDAC6 deacetylase activity, our study demonstrates a functional effect of RanBPM on HDAC6 activity in cell migration and reveals the involvement of the CTLH complex in HDAC6 inhibition. Investigation of the specific domains of RanBPM and HDAC6 that mediate their interaction revealed that, while their interaction does not appear to be direct, the LisH domain of RanBPM is required for association with HDAC6, while HDAC6 interaction with RanBPM requires its second catalytic domain. We show that RanBPM colocalization with α-tubulin is dependent on HDAC6 and that both the LisH and CTLH domains are required for this association. We demonstrate that members of the CTLH complex are able to colocalize with microtubules and inhibit HDAC6 activity and that muskelin is able to associate with HDAC6 independently of RanBPM, suggesting that muskelin may bridge RanBPM to HDAC6. Finally, we show that RanBPM’s association with HDAC6 inhibits HDAC6 activity and prevents HDAC6-mediated cell migration.

A proteomic screen previously demonstrated that muskelin associates with HDAC6 [50]. We confirm here that muskelin does form a complex with HDAC6 through co-immunoprecipitation analysis and show that the HDAC6-muskelin association occurs independently of RanBPM, as muskelin and HDAC6 still associated in RanBPM shRNA cells. We previously showed that RanBPM associates with both HDAC6 and the microtubules, specifically α-tubulin [48], and RanBPM and muskelin have been shown to be associated and be a part of the CTLH complex [5, 13, 51]. Multiple domains of RanBPM are required for the interaction with muskelin, including the LisH domain [51]. In this study we demonstrated that other members of the CTLH complex, namely Twa1 and MAEA, are also localized at microtubules. This suggests that the entire CTLH complex is present at microtubules and associates with HDAC6 through muskelin. Interestingly, there is evidence of other LisH domains mediating interactions with HDAC proteins. Transducing β-like 1 (TBL1) and transducing β-like 1 receptor (TBLR1), LisH domain-containing proteins, were shown to be part of a large protein complex encompassing HDAC3 that functions as a transcriptional repressor. Similar to how the RanBPM association with HDAC6 was significantly reduced upon deletion of the LisH domain, both TBL1 and TBLR1 lose their ability to associate with HDAC3 when the LisH domain is removed [54]. Therefore, it is possible that the LisH domain of muskelin mediates its association with HDAC6, bringing the entire CTLH complex, including RanBPM, in proximity to HDAC6.

Our data demonstrate that RanBPM association with microtubules is mediated through its LisH and CTLH domains. LisH domains have been shown to be important for dimerization and also binding to microtubules [3], however the role of the CTLH domain in microtubule interaction is still unclear. Also, whether muskelin is required for RanBPM to associate with microtubules, via α-tubulin or if this association is dependent on another protein, which would bridge RanBPM and α-tubulin remains to be investigated. As all members of the CTLH complex, with the exception of Armc8, have LisH and CTLH domains [13], it is also possible that other CTLH complex members may function to recruit the CTLH complex to microtubules [48].

Our results suggest that the CTLH complex modulates HDAC6 activity, but how this is achieved remains to be investigated. The obvious conclusion would be that the CTLH complex, through its putative E3 ubiquitin ligase activity, inhibits HDAC6 by targeting it for degradation by the proteasome through the addition of ubiquitin. Recently, RMND5A from *Xenopus laevis* was demonstrated to have E3 ubiquitin ligase activity [55], but whether the mammalian CTLH complex retains E3 ubiquitin ligase activity still remains to be verified. We have previously demonstrated, however, that HDAC6 protein levels remain unchanged in control and RanBPM shRNA cells [47], indicating that RanBPM is not inhibiting HDAC6 activity through modulation of its protein levels. Thus, the CTLH complex activity could potentially affect an HDAC6 regulator, as HDAC6 activity is positively and negatively modulated through many post-translational modification events and interactions with several proteins. HDAC6 activity is positively regulated by phosphorylation by PKCζ, PKCα, GRK2, GSKβ, CK2, ERK and Aurora A and negatively regulated by phosphorylation by EGFR and acetylation by p300 [19–27]. Therefore we speculate that RanBPM’s inhibitory effect on HDAC6 is mediated by the E3 ubiquitin ligase activity of the CTLH complex, which could target an activator of HDAC6 activity for proteasomal degradation.

HDAC6 activity is also modulated through its interaction with other proteins. Association with dysferlin, p62, paxillin, tau and TPPP/p25 have all been reported to result in decreased HDAC6 α-tubulin deacetylase activity [28–32]. Similar to RanBPM, p62 associates with HDAC6 via the second catalytic domain and inhibits its tubulin deacetylase activity, but the precise mechanism of p62 inhibition of HDAC6 has not yet been elucidated [29]. The interaction of TPPP/p25 and HDAC6 was reduced by the presence of tubulin [32], suggesting that HDAC6 associates with TPPP/p25 and tubulin via the same domain. Similarly, dysferlin, a transmembrane protein, inhibits HDAC6 deacetylation of α-tubulin by associating with HDAC6, preventing it from interacting with α-tubulin [28]. However, it does not appear that this is the mechanism through which RanBPM inhibits HDAC6 α-tubulin deacetylase activity, as we observed no significant decrease in the colocalization of HDAC6 and α-tubulin in cells expressing RanBPM shRNA.

HDAC6 retains the ability to associate with α-tubulin even when its catalytic activity is inhibited, either by mutagenesis of critical residues within the catalytic domain or by treatment with an inhibitor [17]. RanBPM is still able to associate with HDAC6 even when HDAC6 is inactivated by chemical inhibition, tubacin treatment or by mutagenesis rendering it catalytically incompetent (Additional file 3: Fig. S3). This indicates that the catalytic activity of HDAC6 is not required for its association with RanBPM.

HDAC6 has been shown to promote cell motility through deacetylation of α-tubulin and/or cortactin. Overexpression of HDAC6 results in increased cell motility, however, overexpression of a catalytically inactive HDAC6 mutant shows motility similar to that of control cells [33]. Consistently, downregulation, knockdown or chemical inhibition of HDAC6 results in reduced cell migration [18, 34–37]. This evidence clearly indicates a role for HDAC6 catalytic activity in promoting cell motility. Interestingly, RanBPM has previously been shown to have a role in inhibiting cell migration. RanBPM was found to inhibit chemotactic migration by associating with leukotriene B4 receptor 2 (BLT2) [56]. Recently, a study performed in gastric cancer cells also reported that downregulation of RanBPM resulted in decreased cell adhesion and increased cell motility [57]. Our own studies also uncovered that cells with stable downregulation of RanBPM had increased migration compared to control cells in a wound healing assay, indicating that RanBPM functions to inhibit cell migration [15]. This led us to evaluate the effect of RanBPM inhibition of HDAC6 on cell migration. RanBPM shRNA cells exhibited increased cell migration when compared to control shRNA and expression of WT RanBPM but not a LisH/CTLH domain RanBPM deletion mutant, which no longer associates with HDAC6, was able to restore cell migration activity to that of control cells. We also demonstrated here that inhibition of HDAC6 with tubacin restored cell migration to that of control cells, indicating that the loss RanBPM modulation of HDAC6 activity is responsible for the increased migration observed in RanBPM shRNA cells. Interestingly, previous studies showed that siRNA-mediated downregulation of muskelin resulted in significantly increased cell migration compared to control siRNA [58], similar to what we observed with stable downregulation of RanBPM. This suggests that the CTLH complex functions to inhibit cell migration and disruption of the complex by downregulation of its components relieves the inhibition of cell migration.

As RanBPM is able to inhibit HDAC6-mediated cell migration, which promotes tumour invasiveness, this identifies a tumour suppressive function for RanBPM. RanBPM has been identified to have other tumour suppressor functions. We previously showed that RanBPM through its inhibition of ERK signaling promotes apoptosis by preventing expression of pro-apoptotic Bcl-2 family proteins at both the transcriptional and protein level [2, 15]. Similarly, RanBPM upregulates the mRNA levels of the pro-apoptotic transcription factor p73, contributing to the activation of apoptosis [59]. Furthermore, downregulation of RanBPM resulted in loss of growth factor dependence as RanBPM shRNA cells were shown to continue to survive and proliferate in the absence of growth serum [15].

Aside from promoting cell migration and consequently tumour invasiveness, HDAC6 contributes to other oncogenic activities. Increased expression of HDAC6 results in deacetylation of Hsp90, enhancing its chaperone activity. Hsp90 client proteins include breakpoint cluster/Abelson murine leukemia viral oncogene homolog 1 (Bcr/Abl), Raf, epidermal growth factor receptor 2 (ErbB2) among others. Many Hsp90 client proteins contribute to cell growth and survival pathways commonly exploited in cancer cells. In fact, Hsp90 inhibitors are also under investigation as chemotherapeutic agents [60]. HDAC6 has also been demonstrated to play a role in promoting angiogenesis, a hallmark of cancer [42, 43, 61]. It would be of interest to evaluate whether RanBPM and its associated CTLH complex are also able to restrict these other oncogenic activities of HDAC6.

## Conclusions

In summary our results demonstrate a functional effect of RanBPM on HDAC6 activity in cell migration and reveal the involvement of the CTLH complex in HDAC6 inhibition. As HDAC6 has been demonstrated to have a causal role in the development of cancer, specific inhibitors have emerged as a promising target for cancer treatment and have been shown to result in decreased cell growth and decreased tumour formation in preclinical studies [44–46]. Since RanBPM has been shown to have tumour suppressor functions and has been demonstrated to inhibit HDAC6 activity, this study provides mechanistic insights to understand the function of RanBPM and its associated CTLH complex in prevention of tumourigenesis and cellular transformation through regulation of HDAC6. Understanding the mechanism by which the CTLH complex is able to inhibit HDAC6 could provide insight on the development of small molecule HDAC6 inhibitors that could be used in chemotherapy treatment.

## Additional files

Additional file 1: Figure S1. Deletion of RanBPM LisH domain prevents inhibition of HDAC6 acetylase activity. Whole cell extracts from RanBPM shRNA Hela cells either left untransfected or transfected with HA-RanBPM-WT or HA-RanBPM-ΔLisH were analyzed by western blot with the antibodies indicated. Right, quantification of relative amounts of acetylated α-tubulin was normalized to total α-tubulin levels. Relative amounts of acetylated α-tubulin in untransfected and HA-RanBPM-ΔLisH-transfected cells were normalized to WT. Results are averaged from three different experiments, with error bars indicating SEM. *P* < 0.05 (*), *P* < 0.005 (**) (PDF 171 kb)

Additional file 2: Figure S2. RanBPM does not directly interact with HDAC6 CAT 2. GST pull-down assays were performed using GST and GST-WT-RanBPM proteins purified on glutathione-agarose beads incubated with extracts from E coli-expressing HDAC6 CAT2. Pull-downs were analyzed by Western blot with antibodies to GST (top panel) and T7 (bottom panel) (PDF 61 kb)

Additional file 3: Figure S3. RanBPM associates with catalytically inactive or Tubacin-inhibited HDAC6. **a** *Left,* whole cell extracts were prepared from HEK cells treated with either DMSO or 10 μM Tubacin for 16 h. Extracts were then immunoprecipitated with either RanBPM or IgG. Immunoprecipitates were analyzed by western blot with an HDAC6 antibody. RanBPM immunoprecipitation was verified using a RanBPM antibody. Input, 5% input extract. *Right,* quantification of relative amounts of co-immunoprecipitated HDAC6 normalized to immunoprecipitated RanBPM. Results are averaged from three different experiments with error bars indicating SEM. *P* < 0.05 (*). **b** *Left* ***,*** whole cell extracts were prepared from HDAC6 knockout MEFs untransfected (−) or transfected with full length (FL) or mutated catalytically inactive (DC) HDAC6 constructs. HDAC6 was immunoprecipitated with a FLAG antibody and immunoprecipitates were analyzed by western blot with a RanBPM antibody. HDAC6 mutant immunoprecipitation was verified using an HDAC6 antibody. Input, 5% input extract. *Right,* quantification of relative amounts of co-immunoprecipitated RanBPM normalized to immunoprecipitated HDAC6. Results are averaged from three different experiments with error bars indicating SEM. *P* < 0.05 (*) (PDF 145 kb)

## Abbreviations

1–503: pcDNA-HDAC6–1-503-FLAG
1–840: pcDNA-HDAC6–1-840-FLAG
BUZ: binder of ubiquitin zinc finger
CAT2: Catalytic domain 2
CK2: Casesin kinase 2
CRA: C-terminal CT-11-RanBPM
CTLH: C-terminal to Lissencephaly type-1 like homology
DC: pcDNA-HDAC6-DC-FLAG
DMEM: Dulbecco’s modified Eagle’s medium
EGFR: Epidermal growth factor receptor
EMT: Epithelial-mesenchymal transition
ErbB2: Epidermal growth factor receptor 2
EV: Empty vector
FBPase: Fructose-1,6-bisphosphatase
FL: pcDNA-HDAC6-FL-FLAG
Gid: Glucose-induced degradation
GRK2: G protein-coupled receptor kinase 2
GSKβ: Glycogen synthase kinase 3 β
HA-1-503: pcDNA-HA-HDAC6–1-503-FLAG
HA-1-840: pcDNA-HA-HDAC6–1-840-FLAG
HCC: Hepatocellular carcinoma
HDAC6: Histone deacetylase 6
Hsp90: Heat shock protein 90
IR: Ionizing radiation
KO: knockout
LisH: Lissencephaly type-1 like Homology
MAEA: Macrophage Erythroblase Attacher
MEFs: Mouse embryonic fibroblasts
OSCC: Oral squamous cell carcinoma
PKC: Protein kinase C
PVDF: Polyvinylidene difluoride
RanBPM: Ran-binding protein M
RING: Really Interesting New Gene
Rmnd5A: Required for meiotic nuclear division homolog A
SPRY: Spla kinase and ryanodine receptor
TBL1: Transducing β-like 1
TBLR1: Transducing β-like 1 receptor
TGF: Transforming growth factor
TPPP/p25: Tubulin polymerization-promoting protein/p25
TSA: Trichostatin A
Twa1: Two-hybrid-associated protein 1 with RanBPM
Vid: Vacuolar import and degradation
WT RanBPM: pCMV-HA RanBPM shRNA mutant construct
WT: Wildtype
Δ360: pCMV-HA-RanBPM- Δ360
ΔCTLH: pCMV-HA-RanBPM- ΔCTLH
ΔLisH: pCMV-HA-RanBPM- ΔLisH
ΔN-439: pcDNA-HDAC6- ΔN-439-1215-FLAG.
