# Supplementary material for: Inhibition of HDAC6 activity through interaction with RanBPM and its associated CTLH complex
Source: BMC Cancer. 2017 Jul 1;17:460. doi: 10.1186/s12885-017-3430-2 (PMC5494137; doi:10.1186/s12885-017-3430-2)
Supplement: Supplementary file 1 — Deletion of RanBPM LisH domain prevents inhibition of HDAC6 acetylase activity. Whole cell extracts from RanBPM shRNA Hela cells either left untransfected or transfected with HA-RanBPM-WT or HA-RanBPM-ΔLisH were analyzed by western blot with the antibodies indicated. Right, quantification of relative amounts of acetylated α-tubulin was normalized to total α-tubulin levels. Relative amounts of acetylated α-tubulin in untransfected and HA-RanBPM-ΔLisH-transfected cells were normalized to WT. Results are averaged from three different experiments, with error bars indicating SEM. P < 0.05 (*), P < 0.005 (**) (PDF 171 kb) [file 12885_2017_3430_MOESM1_ESM.pdf]

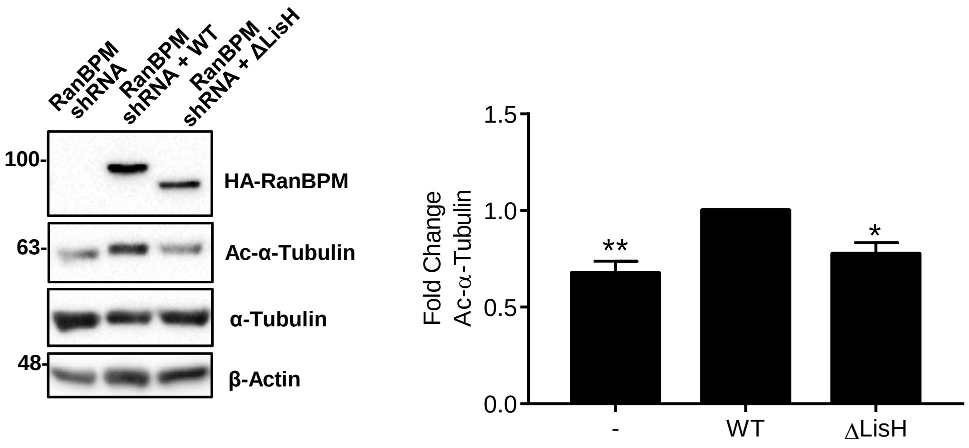

Figure S1. Deletion of RanBPM LisH domain prevents inhibition of HDAC6 acetylase activity. Whole cell extracts from RanBPM shRNA Hela cells either left untransfected or transfected with HA-RanBPM-WT or HA-RanBPM-ΔLisH were analyzed by western blot with the antibodies indicated. Right, quantification of relative amounts of acetylated α-tubulin was normalized to total α-tubulin levels. Relative amounts of acetylated α-tubulin in untransfected and HA-RanBPM-ΔLisH-transfected cells were normalized to WT. Results are averaged from three different experiments, with error bars indicating SEM. $P<0.05$ (*); $P<0.005$ (**).
