# Supplementary material for: Inhibition of HDAC6 activity through interaction with RanBPM and its associated CTLH complex
Source: BMC Cancer. 2017 Jul 1;17:460. doi: 10.1186/s12885-017-3430-2 (PMC5494137; doi:10.1186/s12885-017-3430-2)
Supplement: Supplementary file 2 — RanBPM does not directly interact with HDAC6 CAT 2. GST pull-down assays were performed using GST and GST-WT-RanBPM proteins purified on glutathione-agarose beads incubated with extracts from E coli-expressing HDAC6 CAT2. Pull-downs were analyzed by Western blot with antibodies to GST (top panel) and T7 (bottom panel) (PDF 61 kb) [file 12885_2017_3430_MOESM2_ESM.pdf]

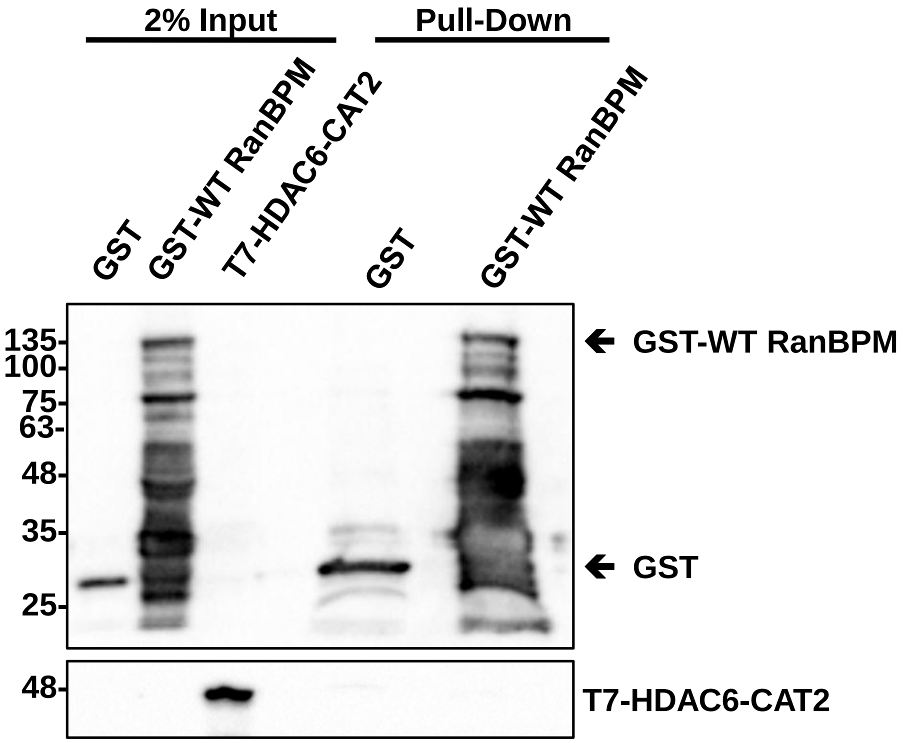

Figure S2. RanBPM does not directly interact with HDAC6 CAT 2. GST pull-down assays were performed using GST and GST-WT-RanBPM proteins purified on glutathione-agarose beads incubated with extracts from E coli-expressing HDAC6 CAT2. Pull-downs were analyzed by Western blot with antibodies to GST (top panel) and T7 (bottom panel).
