# Supplementary material for: Inhibition of HDAC6 activity through interaction with RanBPM and its associated CTLH complex
Source: BMC Cancer. 2017 Jul 1;17:460. doi: 10.1186/s12885-017-3430-2 (PMC5494137; doi:10.1186/s12885-017-3430-2)
Supplement: Supplementary file 3 — RanBPM associates with catalytically inactive or Tubacin-inhibited HDAC6. a Left, whole cell extracts were prepared from HEK cells treated with either DMSO or 10 μM Tubacin for 16 h. Extracts were then immunoprecipitated with either RanBPM or IgG. Immunoprecipitates were analyzed by western blot with an HDAC6 antibody. RanBPM immunoprecipitation was verified using a RanBPM antibody. Input, 5% input extract. Right, quantification of relative amounts of co-immunoprecipitated HDAC6 normalized to immunoprecipitated RanBPM. Results are averaged from three different experiments with error bars indicating SEM. P < 0.05 (*). b Left , whole cell extracts were prepared from HDAC6 knockout MEFs untransfected (−) or transfected with full length (FL) or mutated catalytically inactive (DC) HDAC6 constructs. HDAC6 was immunoprecipitated with a FLAG antibody and immunoprecipitates were analyzed by western blot with a RanBPM antibody. HDAC6 mutant immunoprecipitation was verified using an HDAC6 antibody. Input, 5% input extract. Right, quantification of relative amounts of co-immunoprecipitated RanBPM normalized to immunoprecipitated HDAC6. Results are averaged from three different experiments with error bars indicating SEM. P < 0.05 (*) (PDF 145 kb) [file 12885_2017_3430_MOESM3_ESM.pdf]

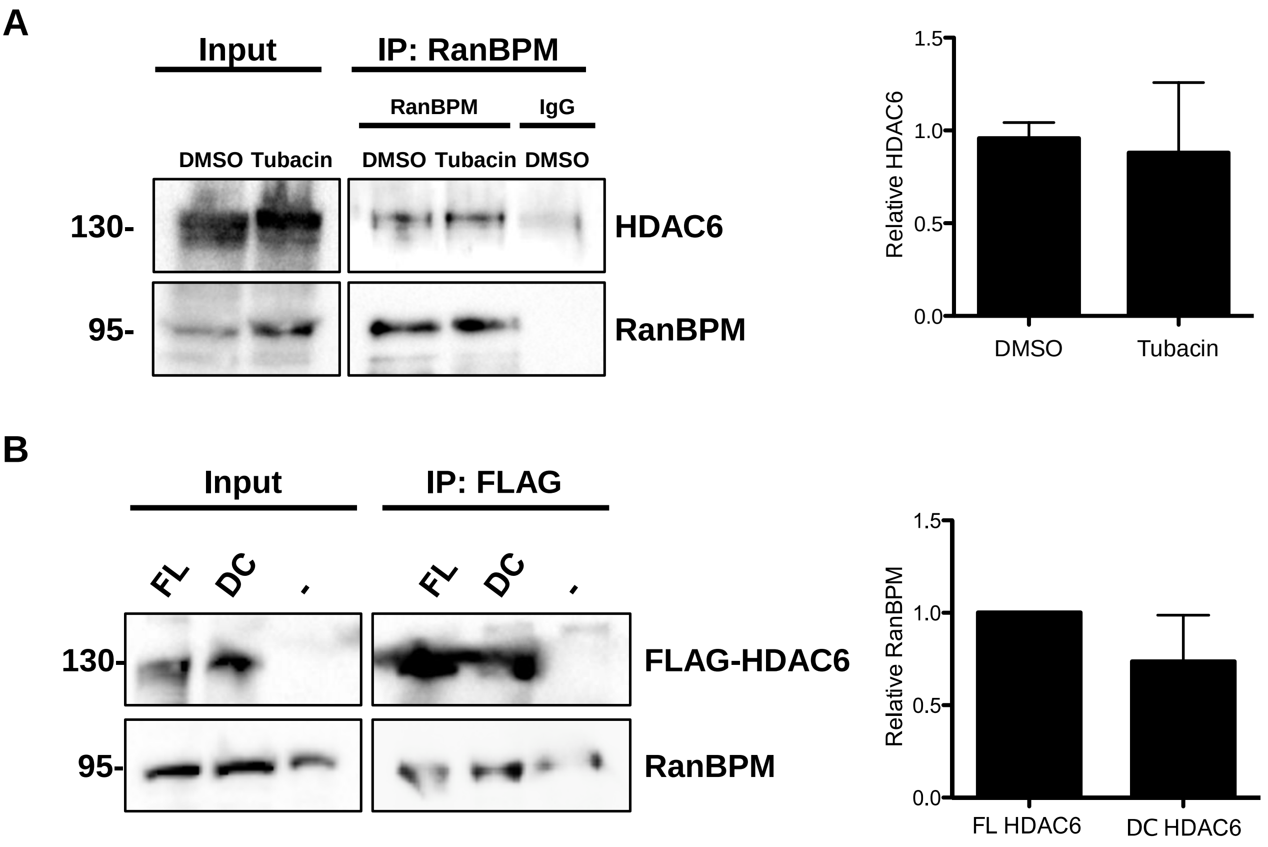


**Figure S3. RanBPM associates with catalytically inactive or Tubacin-inhibited HDAC6**. **a** *Left*, whole cell extracts were prepared from HEK cells treated with either DMSO or 10µM Tubacin for 16 hours. Extracts were then immunoprecipitated with either RanBPM or IgG. Immunoprecipitates were analyzed by western blot with an HDAC6 antibody. RanBPM immunoprecipitation was verified using a RanBPM antibody. Input, 5% input extract. *Right,* quantification of relative amounts of co-immunoprecipitated HDAC6 normalized to immunoprecipitated RanBPM. Results are averaged from three different experiments with error bars indicating SEM. $P<0.05$ (*). **b** *Left*, whole cell extracts were prepared from HDAC6 knockout MEFs untransfected (-) or transfected with full length (FL) or mutated catalytically inactive (DC) HDAC6 constructs. HDAC6 was immunoprecipitated with a FLAG antibody and immunoprecipitates were analyzed by western blot with a RanBPM antibody. HDAC6 mutant immunoprecipitation was verified using an HDAC6 antibody. Input, 5% input extract. *Right,* quantification of relative amounts of co-immunoprecipitated RanBPM normalized to immunoprecipitated HDAC6. Results are averaged from three different experiments with error bars indicating SEM. $P<0.05$ (*).
